# Bioinformatic discovery of microRNA precursors from human ESTs and introns

**DOI:** 10.1186/1471-2164-7-164

**Published:** 2006-07-03

**Authors:** Sung-Chou Li, Chao-Yu Pan, Wen-chang Lin

**Affiliations:** 1 Institute of Biomedical Sciences, Academia Sinica, Taipei 115, Taiwan, Republic of China; 2 Institute of Bioinformatics, National Yang-Ming University, Taipei 112, Taiwan, Republic of China

## Abstract

**Background:**

MicroRNAs (miRNAs) function in many physiological processes, and their discovery is beneficial for further studying their physiological functions. However, many of the miRNAs predicted from genomic sequences have not been experimentally validated to be authentic expressed RNA transcripts, thereby decreasing the reliability of miRNA discovery. To overcome this problem, we examined expressed transcripts – ESTs and intronic sequences – to identify novel miRNAs as well as their target genes.

**Results:**

To facilitate our approach, we developed our scanning method using criteria based on the features of 207 known human pre-miRNAs to discriminate miRNAs from random sequences. We identified 208 candidate hairpins in human ESTs and human reference gene intronic sequences, 52 of which are known pre-miRNAs. The discovery pipeline performance was further assessed using 130 newly updated pre-miRNA and randomly selected sequences. We achieved sensitivity of 85% (110/130) and overall specificity of 49.7% using this method. Because miRNAs are evolutionarily conserved regulators of gene expression, it is expected that their host genes and target genes should have respective phylogenetic orthologs. Our results confirmed that, in certain mammals, the host genes carrying the same miRNAs are orthologs, as previously reported. Moreover, this observation is also the case for some of the miRNA target genes.

**Conclusion:**

We have predicted 208 human pre-miRNA candidates and over 10,000 putative human target genes. Using sequence information from ESTs and introns ensures that the predicted pre-miRNA candidates are expressed and the combined expression transcription information from ESTs and introns makes our prediction results more decisive with regard to expressed pre-miRNAs.

## Background

miRNAs are endogenous non-protein-coding RNAs (ncRNA) of approximately 22 nt [1]. They have the potential to bind to 3' UTRs of mRNAs via sequence complementarity and, with the aid of other cofactors, down-regulate gene expression at the post-transcriptional level [1]. An increasing body of evidence has shown that miRNAs function not only during development [2] but also in disease progression (e.g., cancer oncogenesis) [3]. Viruses also use miRNAs for host cell invasion and to maintain their parasitism [4]. Hence, many scientists have undertaken studies to delineate the physiological functions of miRNAs and such work will be potentiated by the discovery of novel miRNAs.

Direct cloning of miRNAs in cells is often dominated by a few highly expressed miRNAs. In some cases, novel miRNAs have been difficult to detect in cells due to their small size and low abundance [5]. Although many experimental or/and computational procedures have been developed to identify miRNAs, these procedures still have some disadvantages. The traditional biological method, direct cloning, is limited by the need to acquire a large amount of RNA starting material. In addition, a few highly expressed miRNAs usually constitute the majority of the cloned products, and therefore most low-abundance miRNAs are difficult to detect with direct cloning [6]. As an alternative, some investigators have developed bioinformatic algorithms that predict miRNAs from genome sequences. Several studies have reported serial pipelines to predict miRNAs from *Caenorhabditis elegans*, Drosophila, Arabidopsis and human [6-9]. In brief, these studies used various programs to predict [potential hairpin sequences within genomic sequences. These candidate hairpins were then examined by additional filters. Although these procedures have predicted many candidates with the potential to form qualified hairpins, only a limited number of hairpins have been confirmed experimentally on the basis of expression due to the difficulty of large-scale expression validation and low prediction accuracy for expressed miRNAs.

The difficulty in direct cloning of cellular miRNAs may reflect either tissue- or stage-specific expression or may be a consequence of physiological stress in addition to problem of low abundance. Therefore, it is difficult to choose the appropriate biological samples from which to extract miRNAs. Moreover, because miRNAs are short and single-stranded, they are unstable and likely to be degraded soon after tissues or cells are lysed, thereby compounding the difficulties in isolating and detecting these RNAs. On the other hand, bioinformatic discovery of expressed miRNAs may be more successful if expressed transcripts are used for input rather than genomic DNA sequences, because the majority of a genome sequence cannot be transcribed into RNA. Therefore, the use of expressed transcript information offers some advantages for bioinformatic detection of expressed miRNAs.

Our preliminary scan showed that 86 of the 207 known pre-miRNAs are present in the EST and intronic sequence dataset. Lee and colleagues presented the first direct evidence that miRNA genes can be transcribed by RNA polymerase II [10]. Ying and Lin demonstrated that some of the known miRNAs are derived from introns of protein-coding genes [11]. Rodriguez *et al*. found that mammalian miRNAs overlap not only with introns of protein-coding genes but also introns and exons of mRNA-like non-coding RNAs. In addition, they found only a few miRNAs located in protein-coding transcripts [12]. These studies indicate that it is beneficial to use expressed transcript information to extract potential miRNA sequences.

Based on the findings and conclusions of previous reports, we used human reference gene intronic sequences as raw data to predict human miRNAs. In addition, we also analyzed human EST sequences, excluding those encoding proteins, to predict miRNAs originating from independent transcription units distinct from previously annotated genes.

## Results

### Predicting hairpins from human ESTs and intronic sequences using Srnaloop

The most obvious feature of pre-miRNA (precursor of miRNA) is the hairpin structure, which is folded via intramolecular base pairing. We used the program Srnaloop, developed by Grad *et al*. [7], to predict hairpins from raw sequence data. When operating Srnaloop, we used the parameter "-lml 10" to confine possible hairpins to those having a terminal loop ≥ 10 nt based on the report of Zeng *et al*. [13]. We also adjusted many other parameters to optimize the operating results (see Methods). According to our initial survey, there were originally 86 known human pre-miRNAs in our raw data, 60 of them in intronic sequences and 26 in EST sequences, and the combination of parameters we selected was lenient enough to acquire 89.5% (77/86) of these pre-miRNAs. Subsequently, we acquired 1,350,168 candidate hairpins from human ESTs (359,360 hairpins) and intronic sequences (990,808 hairpins) using Srnaloop, and these hairpins were further processed by the Sequence & Structural features filter based on the features of the 207 known pre-miRNAs [14].

### Inferring the locations of mature miRNAs

**Figure 1:**
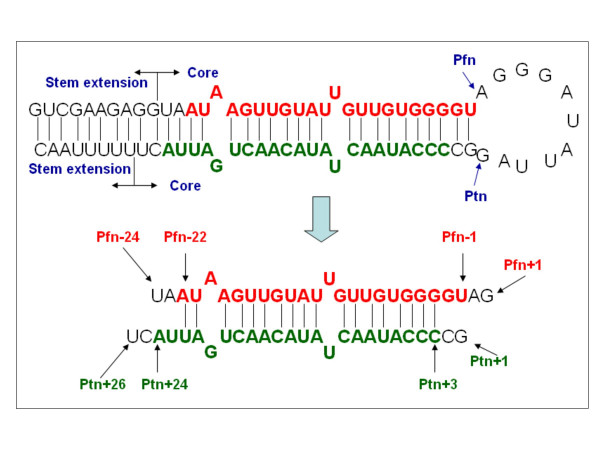
**Illustration of how to infer putative miRNAs**. After applying Srnaloop, we noted the positions of the first nucleotide (Pfn) and the terminal nucleotide (Ptn) of the terminal loop. We elongated each putative miRNA by two nucleotides at each end. By doing so, we acquired 26-nt putative miRNAs, each of which is located between (Pfn-24, Pfn+1) or (Ptn+1, Ptn+26) within each candidate hairpin.

**Figure 2:**
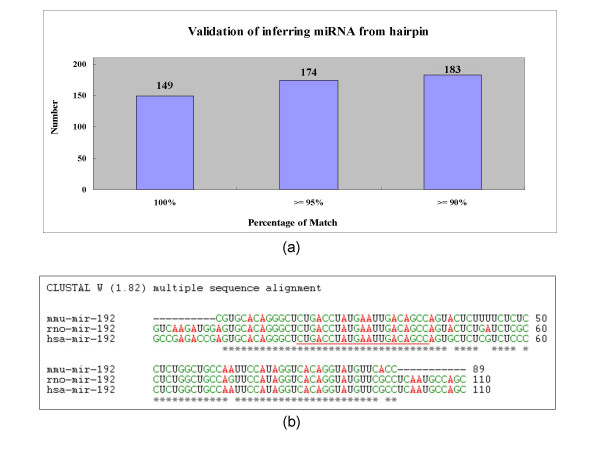
**Evaluation of inferring putative miRNAs and sequence comparison among mir-192 orthologs from distinct species**. (a) We inferred putative miRNAs from 195 known pre-miRNAs (detected by Srnaloop). We then compared the sequences of the known mature miRNAs with those of the putative miRNAs. The results show that 93.8% of the known miRNAs were almost entirely included in the putative miRNAs inferred from their corresponding precursors. This high level of coverage enabled us to use the putative miRNA sequences for the conservation examination. (b) Mir-192 distributes in human (hsa), mouse (mmu) and rat (rno). Using ClustalW [30], we compared sequences of mir-192 orthologs. The alignment shows that most of the mismatches occur in the terminal loop, the opposite arm and the external portion of the hairpins. Besides the mature functional sequences, the entire pre-miRNA sequences are also highly conserved.

The hairpin finding procedure yielded candidate pre-miRNAs in which the candidate mature miRNAs are primarily located in one of the arms of the precursor hairpin. As shown in Figure 1, if Dicer, an RNase III involved in miRNA maturation, precisely cuts the terminal loop at the loop/stem junction, we can infer that the mature miRNAs located approximately at (Pfn-22, Pfn-1) or (Ptn+3, Ptn+24), where Pfn and Ptn represent the positions of the first and terminal nucleotides of the terminal loop, respectively. Given the difficulty in precisely predicting pre-miRNA secondary structure, the actual size of a terminal loop might be larger or smaller, complicating the miRNA boundary prediction. To overcome this problem, we extended the length of each putative miRNA stem sequence by two nucleotides at both ends (22+4). We thus acquired 26-nt putative miRNAs, each of which distributed at (Pfn-24, Pfn+1) or (Ptn+1, Ptn+26) within each candidate hairpin, and was thus named using the suffix "5P" or "3P", respectively. We first tested this boundary prediction strategy by inferring mature miRNAs from 195 known pre-miRNAs containing a hairpin structure that were predicted by Srnaloop from the original 207 miRNA registry entries. As shown in Figure 2a, in 183 of 195 known mature miRNAs, more than 90% of the sequences were identical to the corresponding 26-nt miRNA locations defined here. Among these 183 miRNAs, 149 mature miRNAs were perfectly matched the corresponding putative 26-nt sequences. This information demonstrated that we could obtain useful mature miRNA sequence regions within the candidate pre-miRNAs for subsequent analysis by allowing such wobbling boundary determination, although our study here will not be able to precisely locate the true mature miRNAs.

### Sequence & Structural features filter

There were many fragments that could be predicted to fold into a hairpin structure, but only a few of them represented authentic pre-miRNAs. Therefore, we needed the Sequence & Structural features filter devised based on known miRNA features, to distinguish authentic candidates from false positives. As presented in Table 1, the criteria of the Sequence & Structural features filter are composed of four indices, namely GC content, core minimum free energy (mfe), hairpin mfe and the ratio of core mfe to hairpin mfe (ch_ratio). The definition of these quantifiable features and how to calculate them are discussed in the Methods section.

We first calculated these features, and their distribution ranges are presented in Table 1. After checking the value distribution for each feature, we found that extreme values existed; this required us to widen our criteria, which undoubtedly resulted in an increased number of false positive candidates. Therefore, we narrowed the distribution ranges into reference values and adopted these values as criteria for the Sequence & Structural features filter. As shown in Table 1, the coverage information shows that, even if we excluded these extreme values, the majority of pre-miRNAs still satisfied the selected reference range values. In summary, a candidate for which the GC content, core mfe, hairpin mfe and ch_ratio values were within the individual reference range values was regarded as a positive candidate. Such a combination of criteria resulted in 86.5% (179/207 from known pre-miRNA) sensitivity. In brief, 67,215 candidates predicted from the EST and 113,484 candidates predicted from intronic sequence dataset that survived this filter were further processed by RefSeq filter and conservation examination to find the evolutionarily conserved candidates.

### RefSeq filter

Candidates originating from singleton ESTs, which were without any overlapping with other ESTs, were also processed using the RefSeq filter. We downloaded the human RefSeq sequences [15] with NM accession numbers and used the candidate hairpins to blast search against these protein-coding sequences. Candidates matching protein-coding sequences were removed based on the EST assembly criterion established by The Institute for Genomic Research (TIGR), in which the matching sequence was at least 40 nt in length and had at least 94% identity to be considered part of the identical transcript contig [16]. In brief, 66,109 candidates from the EST and 113,124 candidates from intronic sequence survived this filter.

### Conservation examination and candidates in the Human, Mouse, Dog and Rat (HMDR) dataset

miRNAs have been conserved among phylogenetically close species during evolution. Following the human pre-miRNA discovery pipeline from human ESTs and introns, we searched putative pre-miRNAs against other published mammalian genomic sequences, namely mouse (mm ver. 7), rat (rn ver. 3) and dog (canFam ver. 2) downloaded from the University of California at Santa Cruz (UCSC)[17]. We defined a conserved hairpin as follows: either of the putative miRNAs of a hairpin, 5P or 3P, has a contiguous ≧ 20-nt fragment that is identical to a subject sequence. Both mature miRNAs (arm of hairpin) and pre-miRNAs (entire hairpin structure) are highly conserved (see the case of mir-192 of human, mouse and rat in Figure 2b). After surveying the 207 known human pre-miRNAs to test the reference filter value of this screening approach, we concluded that the entire hairpin must have at least 90% identity with the subject sequence and that the matched sequence must include the 5P, 3P and terminal loop portions in order to be validated.

Using the above criteria, we obtained the human, mouse, dog and rat (HMDR) conservation dataset, and its members represented conserved candidate hairpins having orthologs in human, mouse, dog and rat. Originally, our raw data comprised 842,212 human EST entries and 209,904 intronic sequences. After applying the final conservation filters, there were 208 qualified candidate hairpins in the HMDR set, 52 of which were known pre-miRNAs, resulting in 60.5% (52/86) sensitivity and 25.0% (52/208) specificity (Table 2). Among the 208 qualified candidate hairpins, 61 were located in ESTs and 147 were found in intronic sequences. Each candidate was assigned a unique ID, such as Ih18 or Eh256, in which "Ih" and "Eh" represent a candidate from an intron or EST, respectively.

### Discovery pipeline authentication and specificity assessment

To learn about the efficiency of the pipeline filters used, we first processed the 207 known pre-miRNA (release 5 dataset) using the identical parameters for all procedures in the discovery pipeline. As shown in Table 1, 86.5% of the known miRNAs passed the Sequence and Structural filter (179/207). 62.3% of the known pre-miRNA survival the conservation HMDR examination. Not all 207 pre-miRNAs were present among the input EST and intron sequences; because some of the reported miRNAs are transcribed by polymerase III, they will not be represented in the polymerase II mRNA population. Originally, there were 86 of 207 pre-miRNAs contained in our input sequence data (ESTs and introns). After the RefSeq filter and conservation examination yielded the HMDR set, 69 and 52 pre-miRNAs survived, respectively, implying respective sensitivities of 80.2% and 60.5% for our pipeline. This authenticated our pipeline for discovering putative pre-miRNA candidates from the original polymerase II transcripts (ESTs and introns).

We used two different approaches to assess the prediction specificity for our pipeline. First, we used the updated miRNA registry dataset (release 8)[14] containing 332 known human pre-miRNA records. We extracted the 130 newly added pre-miRNA sequences as our validation test dataset, excluding the 207 pre-miRNAs in the release 5 dataset and some redundant entries. Using the 130 new pre-miRNAs as input query sequence data, we initially detected 116 of the 130 input pre-miRNAs after the initial hairpin finding procedure. We calculated the distributions of quantifiable features, namely GC content, core mfe, hairpin mfe and the ratio of core mfe to hairpin mfe, using the optimized reference values in the discovery pipeline (Table 3). This strategy led to the survival of 110 pre-miRNA entry records. In summary, a sensitivity of 84.6% (110/130) was obtained with the newly added 130 pre-miRNAs in the registry, and this number is similar to the 86.5% listed in Table 1 using the training 207 pre-miRNA dataset (release 5).

The second approach used to assess the pipeline was to use large numbers of randomly selected DNA sequence fragments as negative entries and the 332 known pre-miRNAs (release 8) as positive answers. The validation dataset generation procedure is similar to the one described by Sewer *et al*. [18]. This procedure is based on the fact that the fraction of miRNA-encoding sequences in the human genome is very small; therefore, randomly extracted sequences are extremely unlikely to code for miRNA. In this test, we randomly extracted 99,600 sequence fragments (110 bps in length) from intronic sequences (33,200 fragments), ESTs (33,200 fragments) and genomic sequences (33,200 fragments). The 332 known pre-miRNA sequences and the 99, 600 random sequences of 11 Mbps were applied to our discovery pipeline under the same hairpin finding parameters, Sequence and structural filter and conservation examination between four genomes. Of the 332 known pre-miRNAs, 210 survived the discovery pipeline; this serves as a true positive prediction value. As expected, the number of predicted qualified hairpin candidates from the randomly selected sequences was very small. We obtained 5 total false positives in three independent experiments (2, 1 and 2 predicted candidates, respectively), corresponding to an average of 1.67 false positives in 11 Mbps. Thus, because the initial input EST and intronic sequences are about 1,440 Mbps in length (340 Mbps for ESTs and 1.1 Gbps for introns), we could theoretically generate 212 false positive candidates from similar size dataset. The specificity value of 49.7% was obtained by calculating the percentage ratio of 210 (TP)/(210 (TP) + 212 (FP)) where TP denotes true positives and FP denotes false positives. However, the performance of the discovery pipeline is likely to be better than this value indicates because of the high stringent criteria used in the conservation examination in four genomes, which would reduce the number of randomly generated hairpin sequences.

### Expression levels of candidate miRNAs

Gene Indices developed by TIGR were created by assembling ESTs into virtual transcripts, which were named tentative consensus (TC) records. For the candidates derived from ESTs, the number of ESTs assembled into the host TC records was proportional to the expression level and was therefore used to represent it. Of course, the expression level of each candidate from a singleton EST was assigned as 1. Finally, for candidates derived from introns, we queried the number of ESTs assembled into the UniGene record (Build #190)[19], in which their host genes are clustered. These numbers were used to represent the expression levels of these candidates.

**Figure 3:**
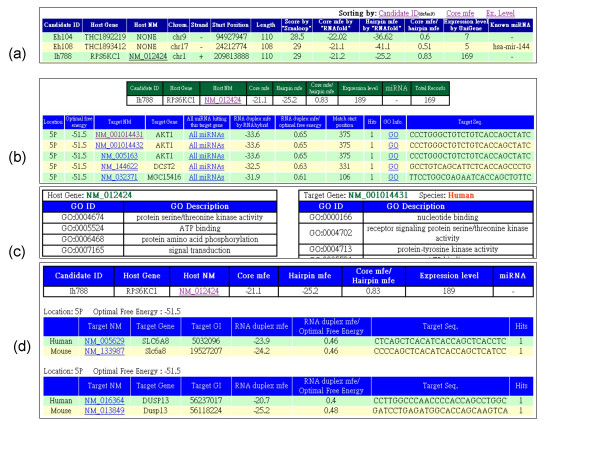
**Illustration of TDL_miRBase dataset report interface**. (a) The complete information report for a candidate hairpin includes host gene, host gene NM accession number (for intronic candidates), genomic location, expression level and match to known miRNAs. The score and minimum free energy (mfe) are the output results from Srnaloop and RNAfold, respectively. (b) Target gene information for a candidate from hairpin Ih788. Target genes were discovered by RNAhybrid and pre-defined conserved motif seeds as described in the text. Optimal free energy and RNA duplex mfe are the output values of RNAhybrid. The GO information of the Ih788 host gene and one of its target genes are displayed in (c). (d) Orthologous target genes report. Some of the target genes were found to be orthologous pairs according to Ensembl gene information. They are displayed as human-mouse or human-rat pairs.

Information for some candidates, including host gene, sequence, genomic location, expression level and so on, is shown in Figure 3a; information for all candidates is available at . The candidates derived from ESTs should be mapped back to the genome and processed to alleviate the redundancy problem (see Methods).

### Using conserved motifs as a bridge to find target genes

miRNAs function as downregulators of genes, and thus a candidate predicted to have target genes has a higher likelihood of being an authentic miRNA. Therefore, we searched for possible target genes of the 208 miRNA candidates in the HMDR set using RNAhybrid [20] and by comparison with conserved motifs [21].

Because miRNAs have been highly conserved in evolution, similar conservation is expected of their targets. miRNAs bind their target sites based on perfect or near-perfect sequence complementarity. Such pairing patterns constrain the variation of miRNAs and their corresponding target sites. Xie and colleagues [21] searched for target sites recognized by miRNAs and compiled 540 conserved and frequent 8-mer motifs by surveying 3' UTRs from human, mouse, rat and dog. We downloaded the 540 conserved motifs and used them to screen our 416 candidate miRNAs (208 × 2; 2 miRNAs per hairpin). We marked a candidate and its corresponding motif as related pairs when the candidate had a contiguous ≧ 6-bp match with a motif.

Next, we identified the 3' UTRs carrying these motifs. From the human reference gene transcript sequence file (human.rna.fna; RefSeq release 3)[22], we retrieved the sequences of 3' UTRs based on the coding sequence positions acquired from the human reference gene genome position file (human.rna.gdff; RefSeq release 3)[22]. We then used these conserved motifs to compare to the 3' UTRs and noted the motifs and their hosting 3' UTRs in pairs. Using the motifs as bridges, we further used RNAhybrid program to align the candidates with the 3' UTRs in pairs, where the target 3' UTRs carried the same motifs matched by the query candidates.

### Using RNAhybrid to find target sites in 3' UTRs

Krek *et al*. used the RNAhybrid program to identify miRNA target genes [20]. They first calculated the optimal free energy of an miRNA when the entire miRNA binds to a perfectly complementary target site. Then they calculated the RNA duplex mfe (minimum free energy of the miRNA/mRNA duplex). We adopted RNAhybrid and its prediction parameters; an alignment for which the RNA duplex mfe was below 33% of its corresponding optimal free energy was regarded as a positive alignment as defined by Krek *et al*. [23]. In addition, based on the conclusion of Lewis [24], the seed match rule was also obeyed under the parameter "-f 2,7", which enforced the criterion that the pairing pattern within the seed must be perfectly matched. Due to our strategy that inferred putative miRNAs, we could not precisely locate the seed within the putative miRNA. Therefore, we adopted an alternative seed match policy, in which a candidate meeting the demand of either "-f 2,7", "-f 3,8", "-f 4,9", "-f 5,10" or "-f 6,11" was considered. Moreover, the matched fragment had to include a conserved motif rather than a random match. In total, we identified 10992 potential targets in all five parameters. There is only a slight difference in the number of targets predicted in each set: 9058 targets (-f 2,7), 9530 targets (-f 3, 8), 9876 (-f 4, 9), 9672 (-f 5, 10) and 9831 (-f 6, 11). The combined predicted targets were almost overlapping in all five sets, indicating that the prediction sensitivity was not affected by the 2-nt shift permitted at the hairpin ends. This might be attributable to the 540 conserved motifs that were used as the bridge for target prediction and to the fact that the prediction was confined within these highly conserved motifs.

Ultimately, there were 155 candidates existed in the HMDR_Target(H) set, in which the candidates were from the HMDR set and were predicted to have specific human target genes (Table 2). Among them, 49 were known pre-miRNAs. Complete information for Ih788 and a partial list of its target genes is presented in Figure 3b, as an example; information on all other human pre-miRNA candidates and their target genes is available at . As shown in Figure 3c, from the "GO information" column, users can access the GO information of Ih788's host gene, NM_012424, and target gene, NM_001014431, in pairs. With this information, we hope to facilitate the study of the interaction between the host gene and target gene at the protein level.

In addition to the human pre-miRNA target genes predicted from human ESTs, we retrieved the sequences of 3' UTRs of mouse and rat genes from the mouse reference gene transcript sequences and rat reference gene transcript sequences (mouse.rna.fna, rat.rna.fna; RefSeq release 3)[22]. Because these putative pre-miRNA candidates are conserved in mouse and rat, we also searched for their target genes in mouse and rat reference genes using the same procedures, and the results can be accessed at . In Table 2, HMDR_Target(H) represents the pre-miRNA candidates found in the HMDR set and also found to have human target genes. HMDR_Target(M) represents the pre-miRNA candidates found in HMDR set and also found to have mouse target genes. HMDR_Target(R) represents the pre-miRNA candidates found in HMDR set and also found to have rat target genes. The identical numbers indicating the highly conserved nature of miRNA and it targets.

### Verifying the conservation of target genes in mammals

Because our candidate miRNAs were conserved among human, mouse and rat, we investigated whether the corresponding target genes that were recognized according to sequence complementarity were also conserved in these species. From Ensembl BioMart[25], we first downloaded the reference tables in which the mouse or rat orthologs of human genes are recorded in pairs. Using the reference tables as bridges, we fetched the target genes in human one by one, and we verified whether the mouse or rat orthologs also corresponded **to **the candidate pre-miRNAs.

According to our results (Table 2), there were 142 candidates in the HMDR_Target(HM) set, in which the candidates were from the HMDR set and their target genes in human and mouse were orthologous genes. Among them, 47 were known pre-miRNAs. Partial orthologous target gene pairs of Ih788 are presented in Figure 3d, and information for all other pre-miRNAs is available at . Moreover, the results for human and rat orthologs are available in Table 1 and . To our knowledge, this is the first report that examines miRNA target genes in orthologous pairs in addition to their host gene conservation.

## Discussion

### Advantages and disadvantages of using intronic and EST sequences as raw data

Our raw data consisted of ESTs and introns rather than genomic sequences. This strategy has some advantages. For instance, other studies have predicted miRNAs using genomic sequences, but it was difficult to confirm their expression. By contrast, most of the ESTs and introns we studied are well annotated, making it easy to acquire information associated with their expression patterns and levels. For example, BX418914 and CF596864, host ESTs containing predicted miRNAs, are expressed in fetal brain and ovary, respectively. Thus, to experimentally confirm these two predicted candidate miRNAs, RNA can be extracted from fetal brain and ovary (or cell lines thereof) rather than from randomly selected tissue types or cell lines.

The disadvantage of this strategy is that the coverage is reduced when ESTs and introns, rather than genomic sequences, are used as raw data. Theoretically, all of the 207 known pre-miRNAs should have matches when searching against the human genome; in our data, however, only 60 and 26 pre-miRNAs matched to introns and ESTs, respectively, implying only 41.5% [(60 + 26)/207] coverage.

### Procedural differences from previous studies

Most pipelines designed to predict miRNAs involve procedures that search for evolutionarily conserved candidates. Grad *et al*. claimed that a conserved candidate had a >20-nt match in subject species and that the matched fragments were located in either arm of the hairpin [7]. The match of only a single arm, however, may not yield sufficient specificity. Besides the miRNA-buried stem, the whole hairpin is also highly conserved. In addition, there are many low-complexity fragments in the genome that may fold into complex RNA secondary structures, including hairpins. Thus, shorter length leads to lower specificity.

In our system, in additon to having conserved miRNAs, the precursor hairpins were required to have more than 90% identity with the subject sequence and that the matched hairpin fragment include the 5P, 3P and terminal loop portions. These criteria ensured that our candidates were phylogenetically conserved miRNAs rather than low-complexity fragments. Our data demonstrate that shorter known hairpins and longer raw sequences produce excellent results that offer an improvement over previous studies.

## Conclusion

We developed our scanning method using criteria based on the features of 207 known pre-miRNAs to predict miRNAs from expressed sequences (ESTs and introns). The statistics demonstrate that our pipeline affords good sensitivity but excellent specificity compared with other published works. In addition to predicting pre-miRNA hairpins, we searched for their target genes in human, mouse and rat. Overall, our results indicate that both the host genes that carry the same putative miRNAs and the target genes recognized by the same miRNAs, are evolutionarily conserved. Finally, we also supply a user-friendly interface to study miRNAs as well as their physiological functions.

## Methods

### Extraction of intronic and EST sequences

We extracted human intronic sequences based on the exonic coordinates recorded in the refFlat.txt file downloaded from UCSC (Build 35)[17]. After parsing the repeat-masked chromosome sequences (hg17)[17] into the array, we could extract the intronic sequences according to the indices derived from previous intron distribution regions. Because many non-miRNA ncRNAs distribute in introns, we masked them by means of RepeatMasker[26], whereby the library is replaced with the ncRNA sequences downloaded from NONCODE [27]. Finally, 209,904 intronic sequences (totaling ~1.1 GB) were extracted for miRNA prediction.

Gene Indices developed by TIGR[28] were created by assembling ESTs into virtual transcripts, named TC records. After annotation, many TC records that were regarded to be part of protein-coding sequences were assigned NP accession numbers, and the numbers of ESTs assembled into each TC record were also noted. Only a few miRNAs are derived from protein-coding sequences [12], and therefore we selected TC records from Human Gene Index (HGI release 14) lacking an NP number and those containing singleton ESTs as our raw data for predicting miRNAs. These selected sequences (~842,212) totaled ~340 MB and were processed by RepeatMasker to identify repeats and ncRNAs before searching for hairpins.

### Srnaloop parameters

Srnaloop calculated the score of each hairpin based on the degree of pairing. The combination of parameters is: "-st r -sml 5000 -Gs -1.5 -lml 10 -gu 1 -t 20 -l 110". The parameter "-st r" means that our input sequence is RNA rather DNA. The parameter "-sml 5000" reflects Srnaloop's capacity for input size. The parameter "-Gs -1.5" means the penalty for a gap initiation is 1.5. The parameter "-gu 1" means the score of a G-U pairing is 1.5. The parameters "-l 110" and "-lml 10" limit the maximum size of the hairpin to 110 and the minimum size of terminal loop to 10 nucleotides, respectively. The predicted hairpins having terminal loop sizes smaller than 10 nucleotides were not missed because Srnaloop was enforced to enlarge their terminal loops to meet the demand of "-lml 10". The parameter "-t 20" limits the score cutoff be 20.

### Quantifiable features used for the Sequences and Structural features filter GC content

Genomes of distinct organisms have different ranges of GC content, and pre-miRNAs are also expected to be so. Thus, we wrote a Perl script to calculate the GC content of each miRNA and determined its reference range value. The distribution of GC content shows that miRNAs are AU rich. For a given putative pre-miRNA, we calculated the GC content of both the putative 5P and 3P miRNAs. As long as the GC content of either putative miRNA located within the reference range value, the pre-miRNA was regarded as a positive candidate.

### Core mfe and hairpin mfe

Generally speaking, a greater a number of paired bases within a hairpin implies greater stability and lower mfe. Srnaloop has no penalty for the terminal loop size, which influences the stability and mfe of the secondary structure owing to steric tension. Therefore, we used RNAfold, a component of the Vienna RNA package [29], to calculate the mfe of each candidate and used mfe as a more precise index for finding authentic miRNAs.

According to the report of Zeng [13], we divided the hairpin structure into two parts, the core and the stem extension. As shown in Figure 1, the core includes the putative RNA duplex plus the terminal loop, encompassing the nucleotides between Pfn-24 and Ptn+26. The stem extension includes the nucleotides that are upstream of Pfn-24 or downstream Ptn+26. We calculated the mfe of the core alone and of the entire hairpin, which includes both the core plus the stem extension, and named these variables core mfe and hairpin mfe, respectively.

### Ch_ratio

As indicated in Table 1, the hairpin mfe has a lower distribution than the core mfe, because the entire hairpin has more paired bases than the core portion. We divided the core mfe by the hairpin mfe, and found that the core contributes more to the hairpin mfe than does the stem extension, and the quotient is referred to as the ch_ratio. We found that the ch_ratio has a fixed distribution range and thus used it as a criterion in the Sequence & Structural features filter.

### Mapping candidates back to the genome and assigning them with unique IDs

Our candidates were derived from either introns or ESTs. For those from ESTs, we had no information related to their locations, so we had to map them back to the genome. To increase precision, we used the original hairpin-carrying ESTs or TCs rather than candidate hairpins to query the human genome. First, we annotated the distribution range of a candidate hairpin within its host EST or TC record. Then, we used these hairpin-carrying sequences to blast against the human genome. A qualified match should completely cover the candidate hairpins. In addition, the query fragments should have more than 94% identity with the subject fragments. Using this strategy, we obtained the qualified subject fragments as well as their location information.

Second, we further used the candidate hairpins to query their corresponding subject fragments with "bl2seq" program from NCBI. The qualified match should also have more than 94% identity with its subject fragment. Pair by pair, we could precisely determine the locations of the hairpins and map them back to the genome. Unexpectedly, we found that many candidate hairpins had more than one qualified match, so we selected the matches with the highest score as their real matches. Hairpins that could not be mapped back to the genome were regarded as false positive candidates and were not considered.

## Authors' contributions

SCL performed pipeline and data mining for miRNA discovery and wrote the draft of this manuscript. CYP was responsible for web interface construction. WCL helped to design the study, guided the work and edited the manuscript. All authors read and approved the final manuscript.

## Figures and Tables

**Table 1:** Distributions and optimal ranges of pre-miRNA quantifiable features. We calculated the distributions of quantifiable features, namely GC content, core mfe, hairpin mfe and the ratio of core mfe to hairpin mfe. Because of the existence of extreme values, we adopted the reference value rather than the original distribution ranges in the Sequence & Structural features filter. This strategy led to 90% coverage and 86.5% sensitivity.

	**GC content**	**Core mfe**	**Hairpin mfe**	**Ch_ratio**
Distribution	21 ~ 68	-42.5 ~ -11.2	-56.1 ~ -24.02	36 ~ 96
Reference value	30 ~ 60	-42.5 ~ -17.0	-50.0 ~ -24.02	50 ~ 96
Coverage	182/195 = 93%	193/195 = 99%	193/195 = 99%	193/195 = 99%

Total coverage	179/195 = 90%	Sensitivity		179/207 = 86.5%

**Table 2:** Statistics of candidates from different result sets. We started with 842,212 ESTs and 209,904 intronic sequences, within which there were originally 26 and 60 pre-miRNAs, respectively. After conservation examination, finding the target, and checking the conservation of target genes, we obtained HMDR, HMDR_Target(H), HMDR_Target(M), HMDR_Target(R), HMDR_Target(HM) and HMDR_Target(HR) sets and calculated the specificity and sensitivity for each set. HMDR are the pre-miRNA candidates conserved in all four genomes (human, mouse, dog and rat). HMDR_Target(H) represents the pre-miRNA candidates found in the HMDR set and also found to have human target genes. HMDR_Target(M) represents the pre-miRNA candidates found in HMDR set and also found to have mouse target genes. HMDR_Target(R) represents the pre-miRNA candidates found in HMDR set and also found to have rat target genes. HMDR_Target(HM) represents the pre-miRNA candidates found in HMDR set and also found to have human and mouse target genes in orthologous pairs. HMDR_Target(HR) represents the pre-miRNA candidates found in HMDR set and also found to have human and rat target genes in orthologous pairs.

	**EST**		**INTRON**		**SUM**			
	
miRNA Candidate Set	Our Candidate	Known miRNA	Our Candidate	Known miRNA	Our Candidate	Known miRNA	Sensitivity	Specificity
HMDR	**61**	**17/26**	**147**	**35/60**	**208**	**52/86**	**60.5%**	**25.0%**
								
HMDR_Target(H)	**45**	**15/26**	**110**	**34/60**	**155**	**49/86**	**57.0%**	**31.6%**
HMDR_Target(M)	**45**	**15/26**	**110**	**34/60**	**155**	**49/86**	**57.0%**	**31.6%**
HMDR_Target(R)	**45**	**15/26**	**110**	**34/60**	**155**	**49/86**	**57.0%**	**31.6%**
								
HMDR_Target(HM)	**39**	**14/26**	**103**	**33/60**	**142**	**47/86**	**54.7%**	**33.1%**
HMDR_Target(HR)	**37**	**15/26**	**93**	**31/60**	**130**	**46/86**	**53.5%**	**35.4%**

**Table 3:** Sensitivity test on 130 newly published pre-miRNAs. We tested the sensitivity of applying the same criteria, derived from the 207 original pre-miRNAs, on 130 newly published pre-miRNAs based on release 8.0. After the hairpin finding procedure, 116 of the 130 input pre-miRNAs were detected. We calculated the distributions of quantifiable features, namely GC content, core mfe, hairpin mfe and the ratio of core mfe to hairpin mfe. Such criteria in the Sequence & Structural features filter led to 85% sensitivity, similar to the result obtained from the test on the original 207 pre-miRNAs.

	**GC content**	**Core mfe**	**Hairpin mfe**	**Ch_ratio**
Distribution	22 ~ 72	-46.1 ~ -6.8	-56.1 ~ -13.3	0.36 ~ 0.96
Reference value	30 ~ 60	-42.5 ~ -17.0	-50.0 ~ -24.02	0.50 ~ 0.96
Coverage	114/116 = 98%	112/116 = 97%	112/116 = 97%	115/116 = 99%

Coverage	110/116 = 95%	Sensitivity		110/130 = 85%
